# Reducing edge loading and alignment outliers with image-free robotic-assisted unicompartmental knee arthroplasty: a case controlled study

**DOI:** 10.1186/s42836-024-00259-x

**Published:** 2024-06-05

**Authors:** Wai Hong Lau, Wai Kiu Thomas Liu, Kwong Yuen Chiu, Man Hong Cheung, Amy Cheung, Ping Keung Chan, Vincent Wai Kwan Chan, Henry Fu

**Affiliations:** 1 Department of Orthopaedics and Traumatology, Division of Joint Replacement Surgery, Queen Mary Hospital, Hong Kong SAR, China; 2 Department of Orthopaedics and Traumatology, Division of Joint Replacement Surgery, The University of Hong Kong, Hong Kong SAR, China

**Keywords:** Unicompartmental knee arthroplasty, Robotic-assisted surgery, Component positioning, Loosening, Early failure

## Abstract

**Background:**

Survivorship of medial unicompartmental knee arthroplasty (UKA) is technique-dependent. Correct femoral-tibial component positioning associates with improved survivorship. Image-free robotic-assisted unicompartmental knee arthroplasty enables preoperative and intraoperative planning of alignment and assessment of positioning prior to execution. This study aimed to compare the radiological outcomes between robotic-assisted UKA (R-UKA) and conventional UKA (C-UKA).

**Methods:**

This retrospective case control study involved 140 UKA (82 C-UKA and 58 R-UKA) performed at an academic institution between March 2016 to November 2020, with a mean follow-up of 3 years. Postoperative radiographs were evaluated for mechanical axis and femoral-tibial component position. Component position was measured by two methods: (1) femoral-tibial component contact point with reference to four medial-to-lateral quadrants of the tibial tray and (2) femoral-tibial component contact point deviation from the center of the tibial tray as a percentage of the tibial tray width. Baseline demographics and complications were recorded.

**Results:**

There was a higher mean component deviation in C-UKA compared with R-UKA using method 2 (17.2% vs. 12.8%; *P* = 0.007), but no difference in proportion of zonal outliers using method 1 (4 outliers in C-UKA, 5.1% vs. 1 outlier in R-UKA, 1.8%; *P* = 0.403). R-UKA showed no difference in mean mechanical alignment (C-UKA 5° vs. R-UKA 5°; *P* = 0.250). 2-year survivorship was 99% for C-UKA and 97% for R-UKA. Mean operative time was 18 min longer for R-UKA (*P* < 0.001).

**Conclusion:**

Image-free robotic-assisted UKA had improved component medio-lateral alignment compared with conventional technique.

**Supplementary Information:**

The online version contains supplementary material available at 10.1186/s42836-024-00259-x.

## Introduction

Unicompartmental knee arthroplasty (UKA) is a commonly performed procedure for patients with isolated medial compartment knee osteoarthritis, with a > 90% patient satisfaction rate [1, 2]. Some reports from high-volume centers have demonstrated that survival rates were more than 90% at 20 years [3–5]. However, the procedure itself is technically demanding, with a higher risk of component malposition compared to total knee arthroplasty (TKA). This may then lead to edge loading, accelerated wear and early loosening.

The advent of robotic-assisted surgery has been shown to reduce surgical error. This is achieved through image-based (preoperative computer tomography), or image-free planning prior to bone cuts. Accurate representation of component position and limb alignment during planning, as well as real-time tracking and feedback during bone cuts are proposed to minimize surgeon error. However, the precise degree of improvement brought about by this technology has not been well quantified in prior studies.

The purpose of this study was to determine if robotic surgery provides quantifiable improvement in medial–lateral component alignment when compared with conventional techniques.

## Materials and methods

This was a retrospective cohort study of 140 patients who underwent medial unicompartmental knee arthroplasty at an academic institution between March 2016 and November 2020, with a mean follow-up period of 3 years and a minimum of 8 months. 58 patients underwent robotic-assisted medial UKA (R-UKA), while 82 received conventional surgery (C-UKA). The allocation of patients to each intervention group was determined by the availability of the robotic system at the time of surgery. Patient inclusion criteria were those with isolated medial compartment osteoarthritis or osteonecrosis of the medial femoral condyle, meeting the indications proposed by Kozinn and Scott. Those with varus deformity of up to 15° were included. Exclusion criteria included those with lateral unicompartmental replacement, TKA, inflammatory arthritis, or suboptimal X-rays.

**Fig. 1:**
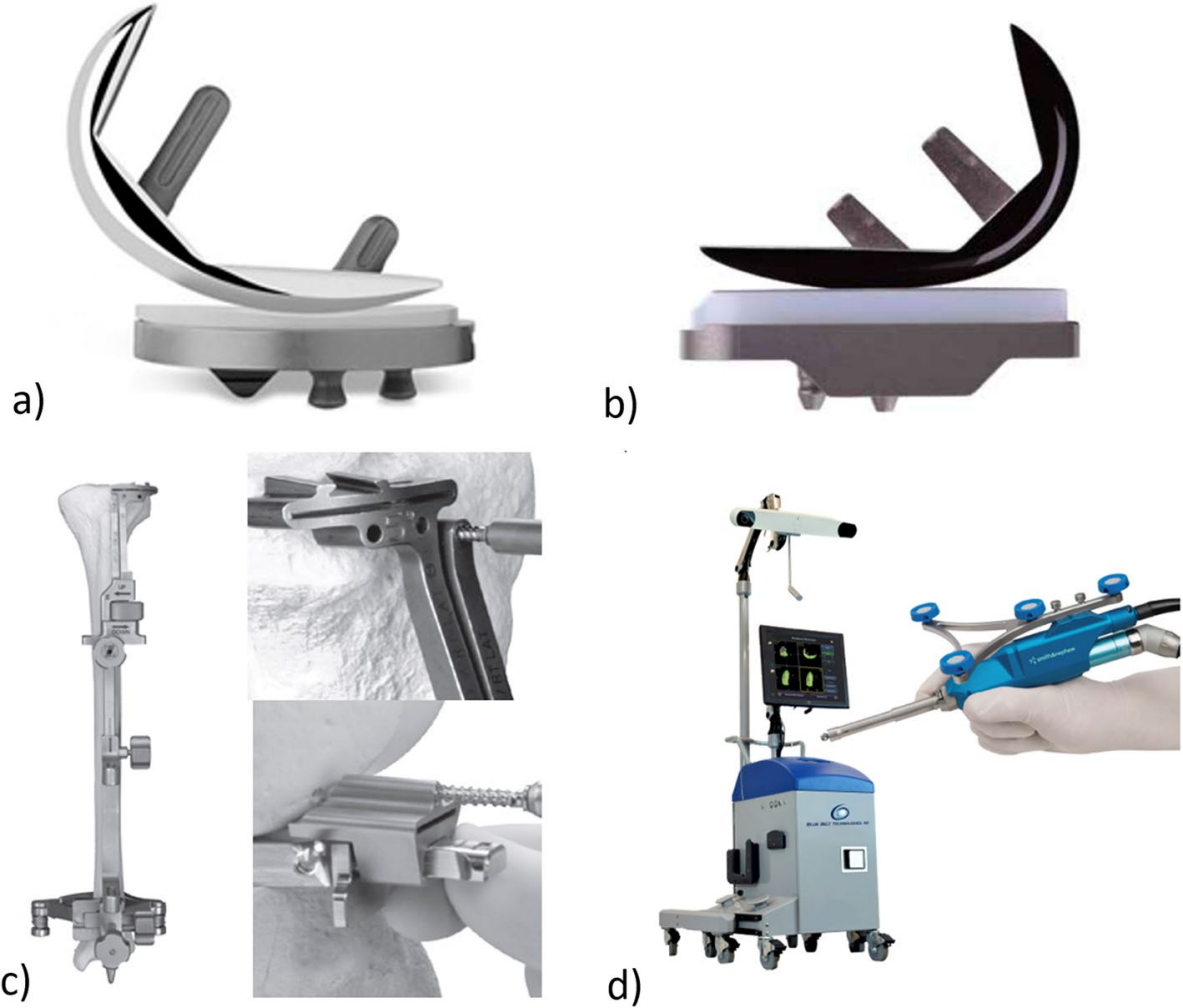
**a** Zimmer ZUK; **b** Journey UNI knee; **c** Conventional UKA instrument; **d** Navio image-free robotic system and hand piece

The surgeries were performed by one of four experienced surgeons at a tertiary referral centre, each with a minimum of 5 years of joint replacement experience and a minimum of 30 UKA procedures per year. All components used in the surgeries were cemented, fixed-bearing, metal-backed on-lay designs. Journey UNI Unicompartmental Knee System (Smith & Nephew, Memphis, TN, USA) was utilized for the robotic group, whereas both the Journey UNI knee system and the Zimmer ZUK system (Zimmer Biomet, Warsaw, IN, USA) were utilized for the conventional group (Fig. 1a-c).

### Surgical technique

The surgical target of both C-UKA and R-UKA was to make the tibial and femoral cuts perpendicular to the mechanical axis and produce an under-corrected varus alignment, typically between 3°–5°. The exact limb alignment was individualized based on the preoperative alignment. Soft tissue releases were minimized with a target laxity of 1–2 mm at final implantation.

In the C-UKA group, all surgeries were performed using a minimally invasive medial parapatellar approach. The surgical steps adhered to the conventional technique and utilized standard instruments as described in the manufacturer’s manual. The procedure involved the removal of medial osteophytes, followed by correct coronal soft tissue balancing of the knee from full extension to deep flexion. Positioning of the femoral component was performed according to patient-specific anatomy, and the tibial component aligned perpendicular to the tibial mechanical axis.

**Fig. 2:**
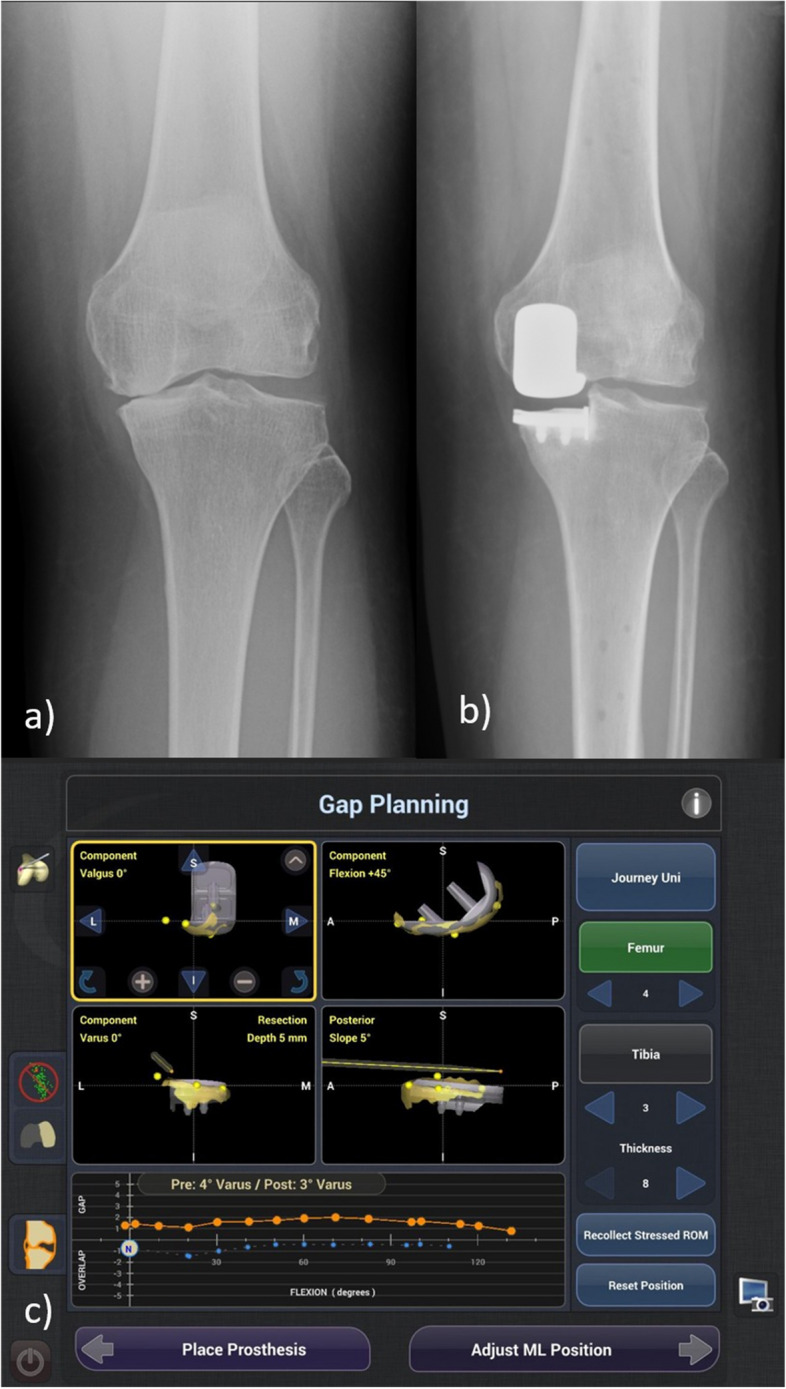
**a** Preoperative X-ray; **b** postoperative X-ray of R-UKA; **c** Navio image-free robotic system intraoperative planning

For the R-UKA group, the Navio image-free robotic system (NAVIO: Journey UNI Unicompartmental Knee System; Smith & Nephew, Memphis, TN, USA) (Fig. 1d) was used. Partially threaded pins were inserted into the proximal tibia and distal femur for the attachment of optical tracking arrays. Osteophytes and loose bodies were first removed. Registration via mapping of the remaining cartilage and bony anatomy was completed in sequence. The optimal tibial slope was determined individually by referencing the lateral intact cartilage as anteromedial cartilage loss is expected in medial OA. Similarly, femoral flexion was matched with the patient’s native anatomy. A soft tissue balancing algorithm was then initiated by applying valgus stress aiming at under-correction of the mechanical axis. Real-time data showing medial laxity were obtained throughout the range of motion, and the individual components were adjusted intraoperatively (allowing up to 3° varus of the tibial component) to produce a medial laxity of 1–2 mm throughout the range of motion. Femoral and tibial component tracking and presence of edge loading were assessed, and component positions were fine-tuned prior to bone removal. A hand-held robotic burr was used to prepare the bone on the condylar surfaces, dynamically modulated by the speed and exposure of the motorized burr tip. After bone preparation, the surfaces were assessed, and trial components were inserted with alignment and soft tissue tension re-assessed. Once the knee was considered properly aligned and balanced, the final components were cemented into place (Fig. 2).

### Outcome measures

Weight-bearing anteroposterior lower limb long-leg radiographs of the knees were taken pre- and postoperatively. All radiographs were taken with the knee fully extended and the knee and foot directed anteriorly. The films that most closely matched an ideal AP knee X-ray, as determined by a proximal tibia-fibular overlap of 1/3 the width of the fibular head, were selected. Lateral X-rays were not analyzed as the primary focus was on coronal component alignment.

**Fig. 3:**
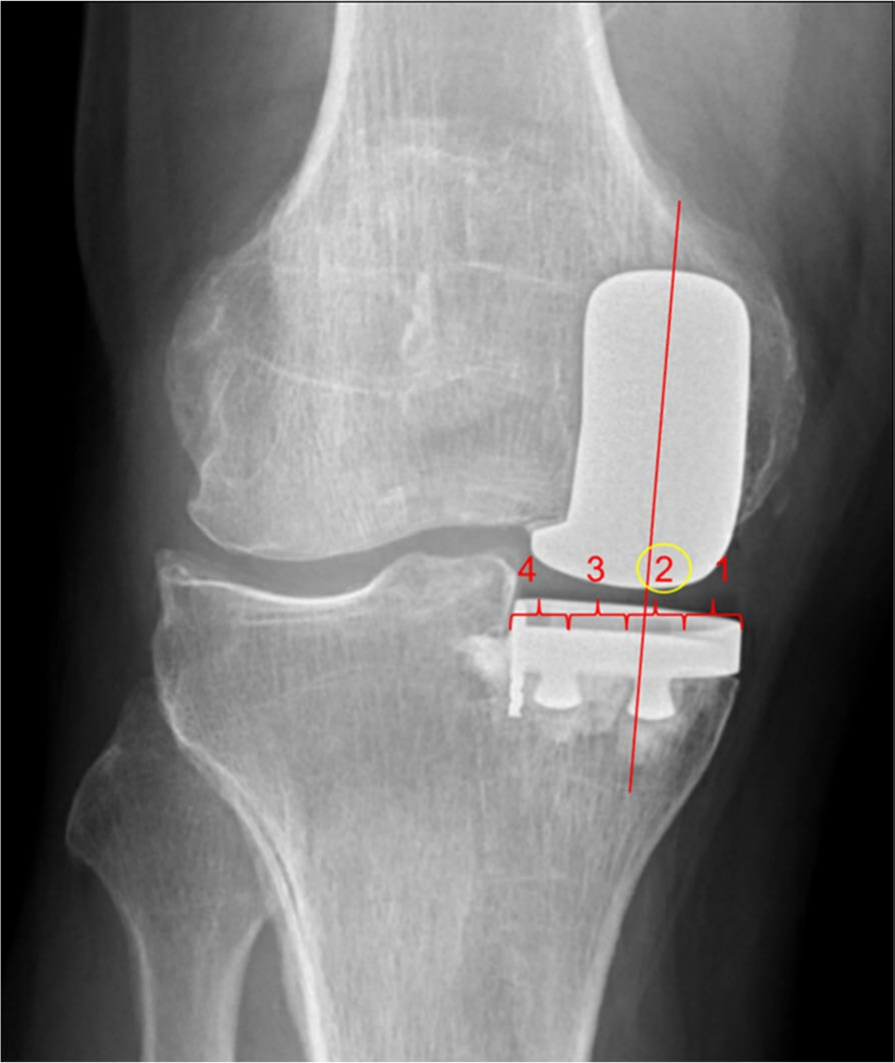
Quadrant method to determine component alignment, recorded as the tibial tray quadrant intersected by the midline of the femoral component

**Fig. 4:**
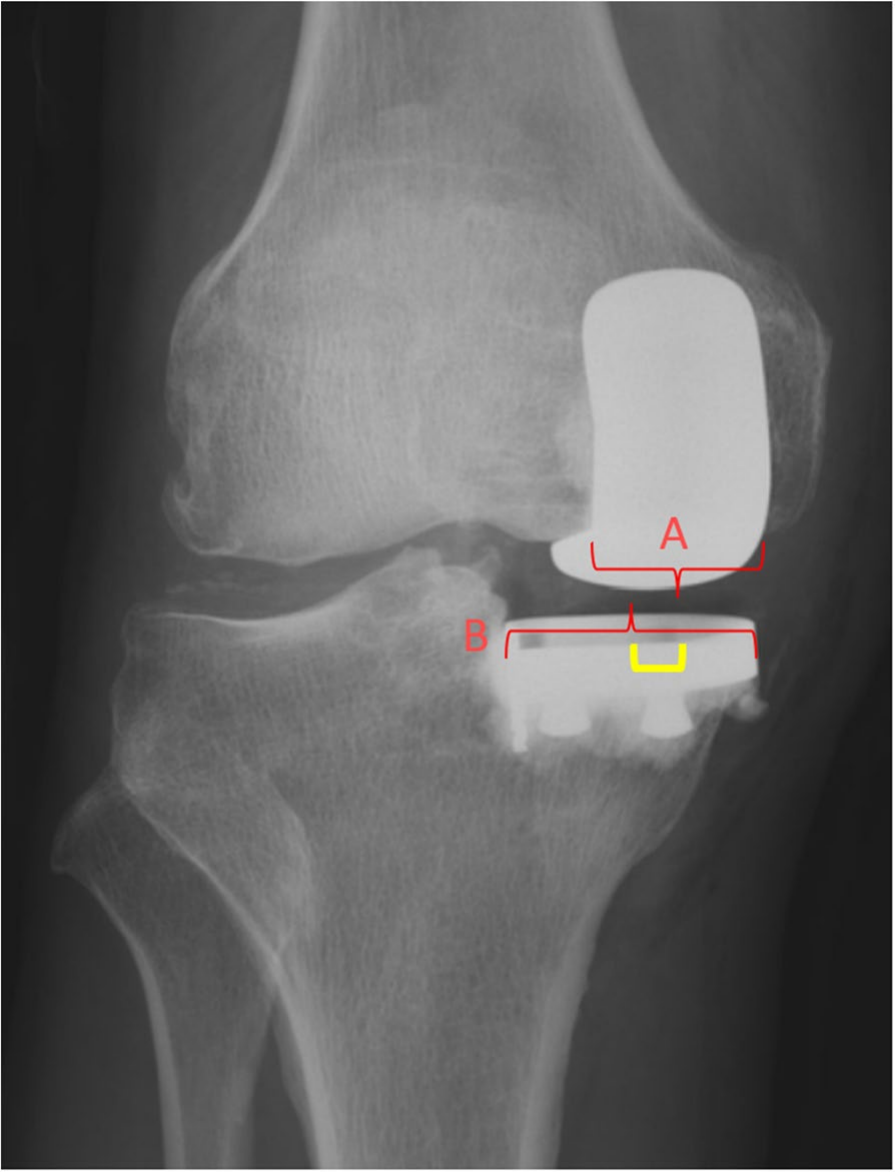
Percentage deviation method to quantify the degree of component alignment

For primary outcome measures, two orthopaedic residents measured the medial–lateral prosthesis positioning using two methods:

1. Quadrant method (Fig. 3): Femoral component midpoint position with reference to four equally-spaced quadrants of the tibial tray. Those with femoral midpoint lying in tibial tray zone 1 & 4 were considered component position outliers, and zone 2 & 3 were deems acceptable.
2. Percentage deviation method (Fig. 4): Deviation between the components were measure by the distance between the midline of the femoral and tibial component (A), divided by the tibial tray width (B) and expressed as a percentage. This was done to account for variance in X-ray magnification.

The measurements were repeated by both residents for the evaluation of intra- and inter-observer errors. Pre- and postoperative limb alignments (Hip-Knee-Ankle angle) were documented. Secondary outcome measures, including postoperative limb alignment, aseptic loosening and duration of operation were documented.

### Statistical analysis

The SPSS statistics software (IBM, Armonk, NY, USA) was used for the statistical analysis. The Student’s *t*-test was employed to compare normally distributed continuous variables, with a significance level of *P* < 0.05 and a 95% confidence interval. The Mann–Whitney U test was used for continuous variables with equal variance that were not assumed (data without a normal distribution). The Chi-square test and the Fisher’s exact test were utilized for the comparison of categorical variables. The inter- and intra-observer variability in measurements on X-rays was determined by the intraclass correlation coefficient (ICC). A range of ICC between 0.75 and 1.00 was considered excellent, indicating the near absence of interobserver variability with a 95% confidence interval. The Cohen’s Kappa coefficient was used for categorical data, with ≤ 0 indicating no agreement, 0.01–0.20 as none to slight, 0.21–0.40 as fair, 0.41–0.60 as moderate, 0.61–0.80 as substantial, and 0.81–1.00 as almost perfect agreement.

Institutional Review Board (IRB) approval was waived due to retrospective nature of this study.

## Results

There were 53 females and 29 males, with an average age of 71 years (range, 50–89 years), and an average body mass index (BMI) of 26.4 ± 3.7 kg/m^2^, in the C-UKA group. In the R-UKA group, there were 46 females and 12 males with an average age of 70 years (range, 51–81), and an average BMI of 25.9 ± 3.4 kg/m^2^. The preoperative mechanical alignment of the operated knees was, on average, 8° ± 5° and 8° ± 4° varus in the C-UKA and R-UKA groups, respectively. The baseline demographics and preoperative mechanical alignment of the two groups were not statistically different (*P* < 0.05). The difference in preoperative Knee Society Knee Score (KSKS) of the two groups was statistically significant, though likely not clinically significant (55 vs. 50). The Knee Society Functional Assessment scores (KSFA) were comparable. Details are outlined in Table 1.

**Table 1:** Patient demographic characteristics

	**Conventional *N* = 82**	**Robotic *N* = 56**	***P* value**
Age (years)	70.9 ± 7.9	69.6 ± 7.3	0.312
Male:Female ratio	1:2	1:4	0.06
Preoperative alignment (varus)	8 ± 5	8 ± 4	0.961
BMI	26.4 ± 3.7	25.9 ± 3.4	0.462
KSKS	55	50	0.009
KSFA	56	57	0.824

Robotic assistance significantly reduced the mean degree of component medial–lateral mismatch in terms of the femoral component midpoint deviation from the midpoint of the tibial component, measured by method 2. There was a mean improvement of 4.4% with the use of robotic assistance (17.2% vs. 12.8%, *P* = 0.007). Details of the results are presented in Table 2. With robotic assistance, there was a tendency towards fewer number of UKAs with component midpoint deviation of more than 20% from midline, shown by Supplementary Graph S1 as a side-by-side comparison bar chart. There was also a tighter interquartile range (6.8%–18% vs. 8.8%–24%) of component midpoint deviation performed with R-UKA compared to C-UKA, shown by Supplementary Graph S2 as a simple box plot.

**Table 2:** Secondary outcome measures

	**Conventional**	**Robotic**	***P*-value**
Outliers vs Non-outliers by Quadrant method	4 vs. 78	1 vs. 57	0.403
Post-operative limb alignment	5° ± 4°	5° ± 3°	0.250
Two-year survivorship	1/82 (99%)	1/57 (97%)	0.916
Operative duration	101 min	119 min	< 0.001
1-year KSKS	92	89	0.996
1-year KSFA	75	78	0.716

The intraclass correlation coefficient (ICC) of measurements for the percentage deviation of the midpoints of the femoral/tibial components were checked for intra-observer and inter-observer variability. The intra-observer ICC was 0.957–0.96, and the inter-observer ICC was 0.974–0.99. This indicated that the degree of intra- and inter-observer error with this measurement method was negligible, and it was a reproducible method of measuring component medial–lateral deviation.

With the quadrant method (method 1), position outliers were determined as those with femoral component midpoint at the extreme zones of the tibial tray. There was no significant difference between the two groups in the number of zonal outliers (1:57 vs. 4:78; *P* = 0.403). The Cohen’s Kappa coefficient value for intra-observer variability was 0.931–1, and that of the interobserver variability was 0.238–0.249. This indicated that though there was a small intra-observer variability, there was a marked disagreement between observers using this method.

There was no difference between the two groups in terms of postoperative limb alignment (5.4 vs. 4.7, *P* = 0.250). There was a tendency toward a higher proportion of patients with ideal correction (1°–3° varus) in the robotic group (conventional 18:40 vs. robotic 14:68), but there was no statistical significance (*P* = 0.121). For complications, there was one case of unexplained pain that ultimately required a late revision to TKA in the R-UKA group.

Two-year survivorship was comparable between the two groups (99% vs. 97%), with one case of aseptic loosening in each group. Both cases were revised to TKA. Operative duration was significantly longer with robotic assistance (101 vs. 119 min, *P* < 0.001). The postoperative 1-year KSKS and KSFA were comparable between the two groups. Operative duration was significantly longer with robotic assistance (101 vs. 119 min, *P* < 0.001). Secondary outcomes are summarized in Table 2.

## Discussion

Up to 96% of patients who undergo UKA have a probability of returning to their preoperative activity levels [6–9]. However, long-term survival remains a significant concern for conventional UKA, despite its good functional outcomes. The revision rates for UKA were at around 4.5% at 2 years in the Australian and Swedish registries, with loosening being the primary cause of revision in patients under 65. At 10 years, survivorship drops to 73%–87%, against 93.3% for TKA [10].

Research indicated that mal-alignment in UKA can impact survivorship [3–5]. Deviation from a safe range of component alignment can increase aseptic loosening risk. Specifically, tibial component coronal mal-alignment beyond 3°, posterior slope exceeding 7° [11], and mechanical limb alignment greater than 5° varus [12–14] have been linked to failure. Diezi et al. highlighted the problem of femoral and tibial component relative mismatch [15]. They found that altering the coronal femorotibial contact angle could quadruple local PE liner stress, leading to accelerated wear and failure. Medial–lateral mismatch may cause lateral tibial subluxation on the femur, potentially leading to loading of the medial edge of the tibial component or lateral femoral condyle impingement on the lateral intercondylar tibial spine [16, 17]. Up to 35% of UKA have significant medial–lateral mismatch [18], which predisposes to edge loading and catastrophic failure. Despite mobile-bearing UKA’s round-on-round bearing geometry (compared to round-on-flat designs of fixed bearing UKA), protecting against edge loading and allowing for a higher degree of component tilting, accurate positioning is still crucial to preventing bearing dislocation due to medial–lateral mismatch [19, 20]. These findings emphasize the importance of accurate component medio-lateral alignment to minimize edge loading and optimize implant survival.

The influence of surgical experience and the learning curve on component mal-alignment in UKA is noteworthy. Data suggest that surgeons performing a minimal volume of 1 to 2 UKA surgeries per annum can have a failure rate as high as 4%. However, an inverse correlation is observed between the surgeon’s experience and the revision rate. Specifically, surgeons performing over 10 UKA surgeries annually demonstrate a revision rate of 2%, which further diminishes to 1% for those performing more than 30 UKA surgeries per year [15, 21, 22].

Despite the proficiency gained with experience, conventional methods still present challenges, with component deviations from the preoperative plan observed in 40%–60% of the components implanted by even the most experienced surgeons [23, 24]. The complexity is amplified when minimally invasive surgical techniques are employed, with studies indicating a broad spectrum of tibial component alignment, ranging from 18° varus to 6° valgus [13, 25]. This highlights the potential advantages of robotic technology in addressing variables such as surgical technique and surgeon experience. Nevertheless, there is a need for more studies that quantify the improvements in component alignment achieved with robotic technology. In a randomized prospective study, Cobb et al. contrasted the outcomes of 13 R-UKAs with 15 C-UKAs [23]. Postoperative CT scans were utilized to ascertain component alignment in the varus-valgus direction. Remarkably, all patients who underwent robotic bone preparation achieved a coronal plane tibiofemoral alignment within 2° of the intended position, a level of precision only attained by 40% of the patients in the conventional group. Lonner et al. also demonstrated a reduced variance in the tibial slope and component varus/valgus alignment from the preoperative goal when robotic assistance was employed in their cohort of 58 UKAs [26]. Conversely, some studies have reported no improvement in component alignment achieved with robotic surgery [27], although each had their own limitations in the study design. Notably, much of the existing research has primarily concentrated on improvement of component varus-valgus alignment and posterior slope with R-UKA. Our study sought to address this gap in the literature by focusing on component alignment in the medio-lateral plane, a critical factor of edge loading.

The current study hypothesized that, compared to conventional manual instrumentation, there would be less medio-lateral mismatch in component alignment in UKA performed with robotic arm assistance. Variability in component medio-lateral mismatch reduced by 4.4% (17.2% vs. 12.8%, *P* = 0.007) in this study, which was in line with previous studies that suggest robotic assistance improved component alignment. The difference in outliers detected by the quadrant method was not significantly different between the two groups (1:57 vs. 4:78; *P* = 0.403). However, the low inter-observer coefficient value of 0.238–0.249 indicated a discrepancy in the zonal categorization among observers. It was hypothesized that this variation could be due to the proximity of some component midpoints to the intersection point between two zones. Therefore, it is likely an inaccurate method of identifying outliers. Regarding postoperative limb alignment, the R-UKA group showed a trend of having fewer outliers, although the difference was not statistically significant. The alignment of the limb was individualized based on the preoperative deformity, which contributed to the heterogeneity of the results. The prosthesis designs used in the study were the Zimmer ZUK and the Smith & Nephew Journey UNI, with ZUK showing survivorship of up to 90% at 14 years, and 98% at 6 years, comparable to our series [28]. While there may be differences in the direction of peg holes and keel design between the two implants, the radius of curvature over the femoral component and tibial insert was similar, the effect on radiographic outcomes was insignificant. In this series, all surgeries were performed by surgeons with reasonable UKA volume, minimizing technique factor as a variable in the outcome for the C-UKA group. While there may be a learning curve for R-UKA, the likelihood of gross component mal-alignment due to inexperience is low, given the image-guided nature of robotic surgery and the surgeons’ familiarity with conventional UKA.

Although measurements of the tibial/femoral contact point assumed comparable X-ray quality among patients, minute differences in the X-ray beam may generate X-rays with variable degrees of rotation in real life, despite best efforts. Tibio-fibular overlap may not be the ideal calibration for standardization owing to differences in patient morphology. This may represent a weakness in the study design. Though computer tomography would be the most accurate modality for assessing component alignment, the high cost and unjustified radiological exposure to patients make it less practical for a large sample size. For identifying outliers that could be at risk of edge loading, however, X-ray measurements were deemed adequate, as they often deviated significantly from the mean. Reproducibility of the percentage deviation method was also excellent, as demonstrated by a high ICC of > 0.9.

While this study, like others, demonstrated a reduction in error and variance of component alignment with robotic assistance, the difference in survivorship between the two groups was not statistically significant. The influence of alignment on function and survivorship post-UKA remains an area of uncertainty. Moreover, the alignment of components in other planes could also significantly contribute to component longevity. Chatellard et al. identified several component mal-alignments that significantly impacted prosthesis survival, including tibial component obliquity exceeding 3°, slope value over 5°, slope change over 2°, and divergence over 6° between tibial and femoral components [21]. Hernigou et al. also discerned an elevated incidence of aseptic loosening associated with a posterior slope exceeding 7°, which was particularly pronounced in cases where the anterior cruciate ligament was absent [11]. Barbadoro et al. [29] discovered that a varus angulation greater than 5° in the tibial component led to an increase in implant micromotion, which could potentially result in loosening. The current study did not consider additional coronal and sagittal alignment profiles due to the limitations of the study design. An optimal study design should incorporate both sagittal and coronal alignment to ascertain the most acceptable criterion for component alignment that minimizes loosening.

While R-UKA is a relatively recent technology, its short- to medium-term survivorship has shown encouraging results. A prospective multicenter study examined the 2-year outcomes of 1007 consecutive patients who underwent R-UKA and reported a worst-case survival rate of 96.0% at an average follow-up of 2.5 years [30]. In a separate retrospective study, a cohort of 128 patients from five institutions was followed for an average of 2.3 years. The study revealed a survivorship rate of 99.2% for the Navio R-UKA [31]. Furthermore, Kleeblad et al. reported a survivorship rate of 97% after following up 432 R-UKAs from four institutions over an average time of 5.7 years [32]. A recent systematic review involving 38 studies demonstrated a survivorship rate of 96% at a 6-year follow-up [33]. These short-term survivorship rates align with the rates reported in the cohort in the present study. However, it’s important to clarify that this study focused on retrospective evaluation of the radiographical results. It did not attempt to correlate these results with survivorship, and, therefore, a detailed survivorship analysis was beyond the scope of this study.

The question of whether image-base or image-free system is superior remains unanswered due to the scarcity of comparative studies. A recent study conducted by DKH Yee et al. in 2023, which included 166 knees, was one of the few that compared the radiological outcomes of image-based and image-free robotic system for TKAs [34]. The study found a slightly higher deviation from the pre-planned posterior slope in the image-based robotic system, and both had differing, but clinically insignificant component varus/valgus alignment. Moreover, it remains unclear whether the results from robotic TKA can be extrapolated to UKAs. Further research is needed to clarify this point.

Cost and increased operation time were additional concerns for R-UKA. Similar studies also showed increased surgical timing of up to 30 min [27]. Cost–benefit analysis was not performed in this study, as a larger sample size and a longer-term follow-up period is required. Further follow-up studies are needed to translate the significance of component alignment to survivorship to justify the cost associated with routine use of robotic technology. Although patients were matched for baseline demographics, a randomized controlled study would be the most accurate way to determine whether robotic assistance enhance the accuracy of performing UKA.

## Conclusion

Robotic-assisted techniques offer potential advantages in improving medio-lateral component alignment of unicompartmental knee arthroplasty. The precise preoperative planning, real-time assessment of ligament balancing and accurate bone preparation provided by robotic systems may help to reduce mal-position and edge loading. The current literature supports the use of robotic assistance in UKR to improve prosthesis alignment, but further research, including long-term studies on survivorship, is needed to establish its role in routine clinical use.

### Supplementary Information

Supplementary Material 1: Graph. S1 Side-by-side comparison bar chart of the results measured by the percentage deviation method.

Supplementary Material 2: Graph. S2 Distribution of results measured by the percentage deviation method, represented with a simple box plot.

## Data Availability

The datasets generated and/or analyzed during the current study are not publicly available due to patient privacy but are available from the corresponding author on reasonable request.

## Abbreviations

UKA: Unicompartmental knee arthroplasty
R-UKA: Robotic-assisted UKA
C-UKA: Conventional UKA
ICC: Intraclass correlation coefficient
BMI: Body mass index
KSKS: Knee Society Knee Score
KSFA: Knee Society Functional Assessment
TKA: Total knee arthroplasty
