# Supplementary figures and images for: Reducing edge loading and alignment outliers with image-free robotic-assisted unicompartmental knee arthroplasty: a case controlled study
Source: Arthroplasty. 2024 Jun 5;6:33. doi: 10.1186/s42836-024-00259-x (PMC11151636; doi:10.1186/s42836-024-00259-x)

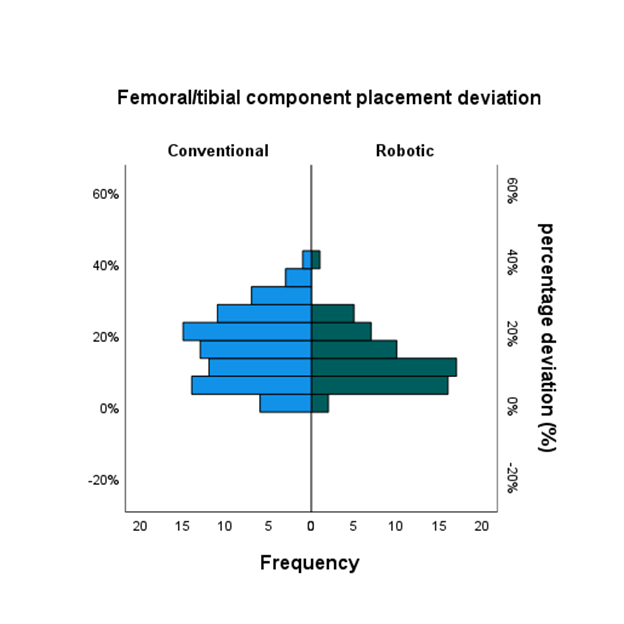

Supplement: Supplementary file 1 — Supplementary Material 1: Graph. S1 Side-by-side comparison bar chart of the results measured by the percentage deviation method. [file 42836_2024_259_MOESM1_ESM.png]

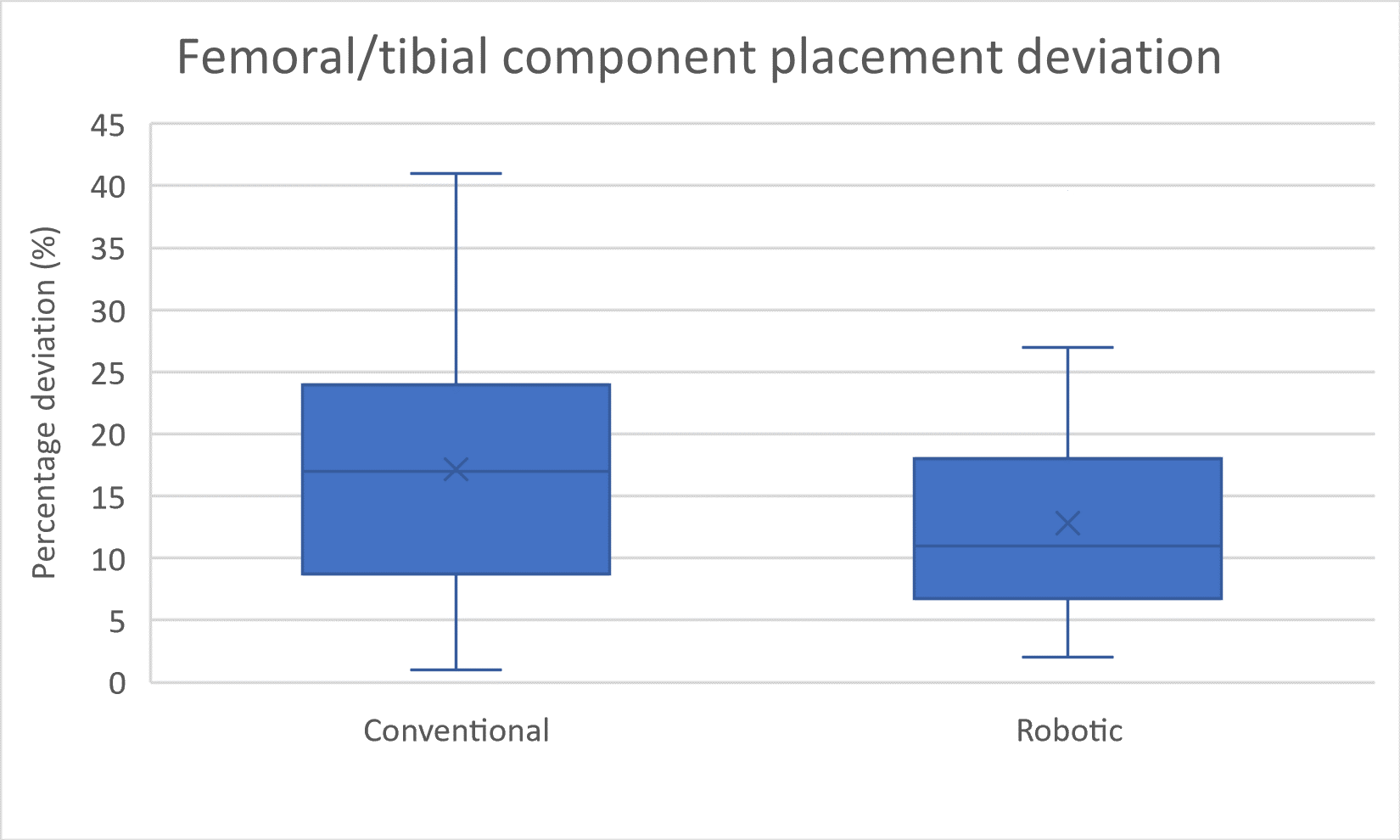

Supplement: Supplementary file 2 — Supplementary Material 2: Graph. S2 Distribution of results measured by the percentage deviation method, represented with a simple box plot. [file 42836_2024_259_MOESM2_ESM.png]
